# The Fusion of Medicine and Surgery

**Published:** 1910-07-16

**Authors:** 


### The Hospital

A Journal of

The Medical Sciences and Hospital Administration.

New Series. No. 179, Vol. VII. [No. 1251, Vol. XLVIII.] SATURDAY, JULY 16, 1910

### The Fusion of Medicine and Surgery.

A well-known gynæcologist has recently remarked that to succeed nowadays in medical practice a young man should be able to "do something." No longer, he said, can a sufficient technical equipment be found in diagnosis and drug dispensing; what the patient has come to look for is ability to supply personally any development in the way of treatment his or her case might demand—*e.g.* the radical cure of a hernia, or the removal of a fibroid tumour.

This *obiter dictum* of Dr. Herman's agrees very well with the gist of a letter to the *Times* of June 17 last contributed by Sir Clifford Allbutt, the parallel being all the closer in that the distinguished Cambridge professor of medicine particularly instances gynæcology, and takes the fairly recent union of medical and surgical practice in this section as an example of what he thinks must happen throughout the field of medicine. His initial argument, from historical precedent, is perhaps not very strong. In the Hippocratic age there was no such division of the art. But what was the intensity of the art then? As well find fault with the encyclopædic Hegel for not rivalling Aristotle's range of attention as expect a single human brain to take all modern medical knowledge as its province! The fate that awaits any ordinary man of talent who attempts this will not be really much better than that of Mr. Panscope, "the chemical, botanical, geological, astronomical, mathematical, metaphysical, meteorological, anatomical, physiological, galvanistical, musical, pictorial, bibliographical, critical philosopher, who had run through the whole circle of the sciences and understood them all equally well." However, this is no doubt quite apparent to Sir Clifford Allbutt, and further on he proposes lightening the task by extending specialism, so that one "physician" should make himself completely efficient in abdominal diseases, another in diseases of the chest, another of the pelvis, and so on. There are the usual arguments against specialism to encounter here, but they will not now be touched upon, partly because, whatever be said, specialism is bound to increase; but mainly for the reason that nothing can be done without a little compromise, and in the matter of bettering the present state of the pure physician something urgently needs doing.

Sir Clifford Allbutt is on strong ground here. "The younger physicians," he says, "are finding out that while they have only permission to undertake the treatment of their cases with one hand tied behind their backs, they are losing them altogether." Indeed patients are not fond of being bandied about from one doctor to another. If a medical man has won their confidence, they prefer him to undertake the whole management of their case himself; and if he is unable to oblige them in this matter, the confidence is apt to be transferred to the one who carries out the requisite treatment. The public, while not appreciating the triumphs of pure diagnosis, soon notices that physicians have to refer patients for treatment to surgeons and specialists more and more every year. Accordingly the young physician suffers unfairly. Sir Clifford Allbutt's remedy is to free his hand, to do away with the old artificial dichotomy of medical and surgical practice, substituting a natural subdivision of labour which corresponds to the regional subdivision of the human body.

The proposal sounds well, and probably does not involve any of the evils commonly urged against over-specialism, although for an instant there suggests itself the witty remark of a general practitioner, who inquired what the "poor umbilicus had done," to be left without a specialist. Not from the patient's side, but from the practitioner's, comes the real objection. And in these days, when written comment is so mealy-mouthed and discriminating, it is hard to name it without the appearance of bluntness. But still the plain fact of the matter is that even among those whose nerves are strong enough for a career in our profession only a small minority have the courage—not of personal antagonism, but of self-exposure to the risk of terrible failure—to enable them to become successful surgeons, and to remain such after forty years of age. To the born surgeon human tissues are merely material for the exercise of his craft, and he handles them with the easy mastery with which a good engineer repairs complicated machinery. Of Sir Clifford Allbutt's "medical thinkers" and "medical bookmen," probably a good number could not make the smallest incision without a certain inward wincing. Many, too, would not be neat-handed enough for life-work in a laboratory. Yet the thinker and the bookman are just as necessary as the craftsmen. What a debt is owing to Sir Patrick Manson's hypotheses! What dozens of papers appear in the medical journals which are both inadequate and superfluous! So long as nature continues to dole out her talents for the most part separately, giving to one character, to another intellect, and so long as real success depends upon natural endowment, so long will the main line of division in medical practice lie between medicine and surgery.

